# Urinary Arsenic Speciation in Children and Pregnant Women from Spain

**DOI:** 10.1007/s12403-016-0225-7

**Published:** 2016-08-12

**Authors:** Antonio J. Signes-Pastor, Manus Carey, Jesus Vioque, Eva M. Navarrete-Muñoz, Cristina Rodríguez-Dehli, Adonina Tardón, Miren Begoña-Zubero, Loreto Santa-Marina, Martine Vrijheid, Maribel Casas, Sabrina Llop, Sandra Gonzalez-Palacios, Andrew A. Meharg

**Affiliations:** 1 Institute for Global Food Security, Queen’s University Belfast, David Keir Building, Malone Road, Belfast, BT9 5BN Northern Ireland, UK; 2 Universidad Miguel Hernández, Avenida de Alicante KM 87, 03550 Sant Joan d’Alacant, Spain; 3 Servicio de Pediatría Hospital San Agustín, Asturias, Avilés, Spain; 4 University of the Basque Country, Bizkaia, Spain; 5 Public Health Department, Basque Goverment, Biodonostia Research Institute, San Sebastian, Spain; 6 ISGlobal, Center for Research in Environmental Epidemiology (CREAL), Barcelona, Spain; 7 FISABIO–Universitat deValència–Universitat Jaume I Joint Research Unit of Epidemiology and Environmental Health, Valencia, Spain; 8 Spanish Consortium for Research on Epidemiology and Public Health (CIBERESP), Madrid, Spain; 9 University Pompeu Fabra (UPF), Barcelona, Spain

**Keywords:** Arsenic speciation, Children, Pregnant women, Biomarker, Urinary metabolites, Inorganic arsenic

## Abstract

Inorganic arsenic (i-As) is a non-threshold human carcinogen that has been associated with several adverse health outcomes. Exposure to i-As is of particular concern among pregnant women, infants and children, as they are specifically vulnerable to the adverse health effects of i-As, and in utero and early-life exposure, even low to moderate levels of i-As, may have a marked effect throughout the lifespan. Ion chromatography-mass spectrometry detection (IC-ICP-MS) was used to analyse urinary arsenic speciation, as an exposure biomarker, in samples of 4-year-old children with relatively low-level arsenic exposure living in different regions in Spain including Asturias, Gipuzkoa, Sabadell and Valencia. The profile of arsenic metabolites in urine was also determined in samples taken during pregnancy (1st trimester) and in the children from Valencia of 7 years old. The median of the main arsenic species found in the 4-year-old children was 9.71 μg/l (arsenobetaine—AsB), 3.97 μg/l (dimethylarsinic acid—DMA), 0.44 μg/l (monomethylarsonic acid—MMA) and 0.35 μg/l (i-As). Statistically significant differences were found in urinary AsB, MMA and i-As according to the study regions in the 4-year-old, and also in DMA among pregnant women and their children. Spearman’s correlation coefficient among urinary arsenic metabolites was calculated, and, in general, a strong methylation capacity to methylate i-As to MMA was observed.

## Introduction

Arsenic (As) is a ubiquitous metalloid that is found in both inorganic arsenic (i-As) and organic forms, which can readily cross the placenta leading to foetal exposure (Concha et al. 1998; Vahter 2009; Davis et al. 2014). Organic As, including arsenobetaine (AsB), arsenosugars and arsenolipids, are often found in fish and seafood and considered relatively non-toxic (Navas-Acien et al. 2011), while i-As, mainly found as arsenite and arsenate, has been classified as a group I, non-threshold, human carcinogen (IARC 2004). Other health effects have also been attributed to i-As exposure such as neurological, cardiovascular, respiratory and metabolic diseases. Exposure to i-As is of particular concern among pregnant women, infants and children, as they are specifically vulnerable to the adverse health effects of i-As, and in utero and early-life exposure, even low to moderate levels of i-As, may have a marked effect throughout the lifespan (Farzan et al. 2013; Davis et al. 2014; Farzan et al. 2015; Gilbert-Diamond et al. 2016; Sanchez et al. 2016). However, further research on links between dietary factors and biomarkers of As is required from areas with relatively low-As exposure (Farzan et al. 2013; Kordas et al. 2016).

Rice, is by far, the main dietary sources of i-As, when low i-As drinking water is available (Sohn 2014; Kippler et al. 2016). The higher levels of i-As in rice compared to other crops are due to anaerobic paddy field culture, which renders i-As highly available for rice plant uptake (Meharg and Zhao 2012). Significantly high levels of i-As have also been found in rice-based products widely consumed by infants and young children (Signes-Pastor et al. 2016b), which have been categorised as a particular sub-population that is more highly exposed to i-As due to higher food consumption rates on a body weight basis than adults (EFSA 2009). Urinary As excretion is a biomarker of the dietary As exposure and a significant increase in urinary As levels has been reported for adults, pregnant women, infants and children after rice consumption (Cascio et al. 2011; Gilbert-Diamond et al. 2011; Davis et al. 2012; Meharg et al. 2014; Karagas et al. 2016).

**Fig. 1:**
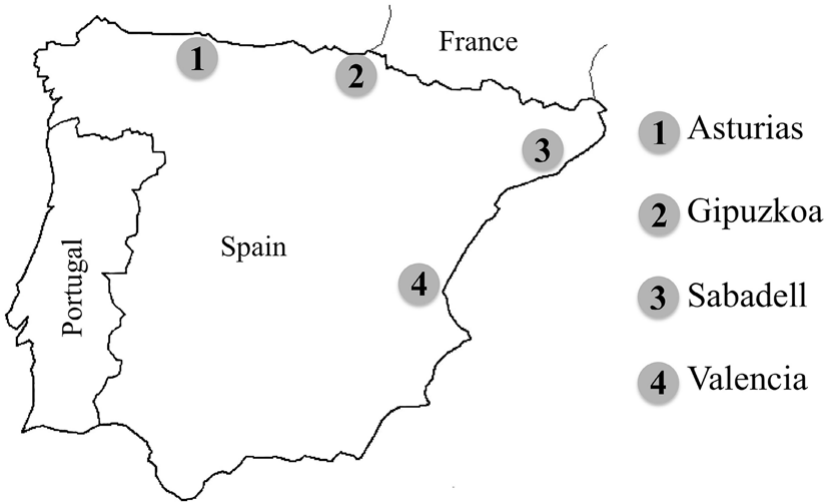
Geographical location of the cohorts in Spain included in this study

In this study, urinary As metabolites were analysed by ion chromatography with inductively coupled plasma—mass spectrometric detection (IC-ICP-MS) and their relationship was evaluated in a study population of 4-year-old children from Spain living in Asturias, Gipuzkoa, Sabadell and Valencia (Fig. 1). The differences in As species concentration in 4-year-old children’s urine samples due to sex and living region were explored. Furthermore, As species and their relationship was studied in urinary samples of pregnant women—children pairs from Valencia, urinary samples of which were collected and evaluated in 4- and 7-year-olds. Likewise, the differences in As species concentrations in urinary samples from pregnant women and their children of 4 and 7 years old were explored.

## Materials and Methods

### Study Population

The population included in the study are mother–child pairs participants in the INMA—*INfancia y Medio Ambiente*—Environment and Childhood project, a prospective population-based birth cohort study conducted in several areas of Spain, www.proyectoinma.org (Guxens et al. 2012). Women were recruited at the beginning of their pregnancy (2003–2006) and were followed up until delivery (*n* = 2625). Their children were enrolled at birth and were followed up during infancy. In this study, a subset of 100 urinary samples from 4-year-old children were randomly (evenly distributed between boys and girls) selected from each study area: Asturias, Gipuzkoa, Sabadell and Valencia. Additionally, urinary samples of the same children from the Valencia cohort were selected at 7 years (*n* = 100) and paired with urinary samples of their mother at 1st trimester of pregnancy (*n* = 100).

Most of the participants in the study here were living in urban (81 %) and semiurban areas (16 %) and only 3 % were living in rural locations. 4-year-old children had a median BMI of 15.99 ranging from 11.52 to 24.99. The median age of the pregnant women was 31 ranging from 21 to 43 years old and 96 % of them have Spanish nationality.

### Sample Preparation

The urine samples were stored at or below −20 °C until analysed. Urinary samples were centrifuged with a Sorvall Legend RT at 4500 g and diluted as appropriate before transferring a 1 ml aliquot to a 2-ml polypropylene vial with 10 μl of analytical grade hydrogen peroxide to convert any arsenite to arsenate to facilitate subsequent chromatographic detection. The urine samples were analysed in different batches including blanks and replicate samples of the certified reference material (CRM) ClinChek^®^—Control level I. Urinary samples were normalised for urine dilution using specific gravity measured with a clinical refractometer. Specific gravity measurement is suggested to normalise urine dilutions since urine creatinine may vary with As-related kidney effects, age and other factors (Carlin et al. 2015).

### Chemical Analyses

A Thermo Scientific IC5000 ion chromatography system, with a Thermo AS7, 2 × 250 mm column and a Thermo AG7, 2 × 50 mm guard column interfaced with a Thermo ICAP Q ICP-MS in collision cell mode was used to determine As speciation in urinary samples. A linear gradient mobile phase was carried out over 15 min starting at 100 % mobile phase of 20 mM ammonium carbonate and finishing at 100 % mobile phase of 200 mM ammonium carbonate. Authentic standards of AsB, DMA, tetratmethylarsonium, MMA and i-As were compared with the resulting chromatogram. DMA concentration series were used to calibrate the As present under each chromatographic peak.

### Statistical Analyses

A descriptive of the different As species was first performed. A Kruskal–Wallis non-parametric test was used to determine any significant differences in the As species levels between groups according to region, sex and population category (pregnant women, 4- and 7-year-old children). Spearman’s correlation coefficient (Rho) was determined to evaluate the relationship among As species logarithm concentration (log_10_), and the LOESS (local regression) method was used for non-linear regression smoothing. All statistical analyses and plots were performed using the R Statistical Software (R Core Team 2014). The limit of detection (LOD) was calculated as the mean of the blank concentrations plus three times the standard deviation of the blank concentrations multiplied by the dilution factor. The ½ LOD value was assigned for statistical analyses of the data when samples were below the LOD.

## Results

### CRM Recoveries

The mean ± SE concentration and recovery, calculated using the mean certified values, of the As species found in the urine CRM ClinChek^®^—Control level I, based on *N* = 33, were as follows: 5.27 ± 0.09 μg/l and 115 ± 2 % for i-As, 2.44 ± 0.04 μg/l and 97 ± 2 % for MMA, 9.19 ± 0.17 μg/l and 94 ± 2 % for DMA and 15.17 ± 0.25 μg/l and 90 ± 2 % for AsB. The mean and range concentrations of the As species certified in the urine CRM ClinChek^®^—Control level I are as follows: 4.55 (2.73–6.37) μg/l for i-As, 2.50 (1.50–3.50) μg/l for MMA, 9.80 (5.88–13.7) μg/l for DMA and 16.8 (12.6–21.0) μg/l for AsB. The limit of detection (LOD) for As speciation, calculated from DMA calibration, was 0.011 μg/l.

### 4-Year-Old Children

The main arsenic species in urine samples of 4-year-old children from Spain were AsB, DMA, MMA and i-As. AsB was predominant with median of 9.71 μg/l and inter-quartile range (IQR) from 2.58 to 34.92 μg/l followed by DMA (median: 3.97 μg/l and IQR: 2.06–6.20 μg/l), MMA (median: 0.44 μg/l and IQR: 0.25–0.69 μg/l) and i-As (median: 0.35 μg/l and IQR: 0.21–0.56 μg/l). There were no significant statistical differences in As species concentration between 4-year-old boys and girls. Statistically significant differences were observed for AsB, MMA and i-As according to the study area at 4-year-old. Children living in Gipuzkoa had the lowest i-As concentration with a median of 0.22 μg/l and IQR from 0.13 to 0.37 μg/l (*p* < 0.001), whereas they had the highest concentration of MMA (median: 0.52 μg/l and IQR: 0.37–0.79 μg/l; *p* < 0.001) and AsB (median: 16.32 μg/l and IQR: 6.25–54.64 μg/l; *p* < 0.001). The region of origin did not affect DMA concentration in the urine samples of 4-year-old children (*p* = 0.131) (Table 1).

**Table 1:** Arsenic speciation (median [25–75 %ile]) in urinary pregnant women, 4-year-old and 7 year-old children samples from each cohort study

Category	N	AsB (μg/l)	DMA (μg/l)	MMA (μg/l)	i-As (μg/l)
Children—4 years	400	9.71 (2.58–34.92)^A^	3.97 (2.06-6.20)	0.44 (0.25-0.69)	0.35 (0.21-0.56)
Girls—4-year-old	200	9.21 (2.44-28.25)	3.82 (1.85-6.13)	0.40 (0.24-0.69)	0.32 (0.21–0.57)
Boys—4-year-old	200	10.51 (2.89–40.41)	4.07 (1.85–6.27)	0.45 (0.25–0.70)	0.37 (0.21–0.56)
*P* value		0.778	0.376	0.568	0.796
Asturias—4-year–old	100	9.07 (1.68–25.31)^b^	3.76 (2.19–5.67)	0.35 (0.18–0.60)^b^	0.38 (0.25–0.54)^a^
Gipuzkoa—4-year-old	100	16.32 (6.25–54.64)^a^	4.23 (2.27–8.92)	0.52 (0.37–0.79)^a^	0.22 (0.13–0.37)^b^
Sabadell—4-year-old	100	5.72 (1.74–20.49)^b^	3.73 (1.62–5.45)	0.49 (0.25–0.84)^ab^	0.39 (0.23–0.65)^a^
Valencia—4-year-old	100	9.01 (2.43–48.78)^ab^	4.19 (2.34–6.39)	0.36 (0.22–0.60)^b^	0.44 (0.28–0.59)^a^
*P*-value		<0.001	0.131	<0.001	<0.001
4-year-old-valencia	100	9.01 (2.43–48.78)	4.19 (2.34–6.39)^b^	0.36 (0.22–0.60)	0.44 (0.28–0.59)
7-year-old-valencia	100	6.81 (1.63–27.61)	4.14 (2.57–6.49)^b^	0.35 (0.22–0.50)	0.40 (0.23–0.57)
Pregnant women-valencia	100	11.96 (4.14–50.34)	5.69 (2.93–10.89)^a^	0.32 (0.19–0.63)	0.42 (0.27–0.62)
*P*-value		0.115	0.003	0.594	0.518

^A^Median (25–75 %ile); values with the same letters were not significantly different at *p*-value <0.05 for the variable studied

**Fig. 2:**
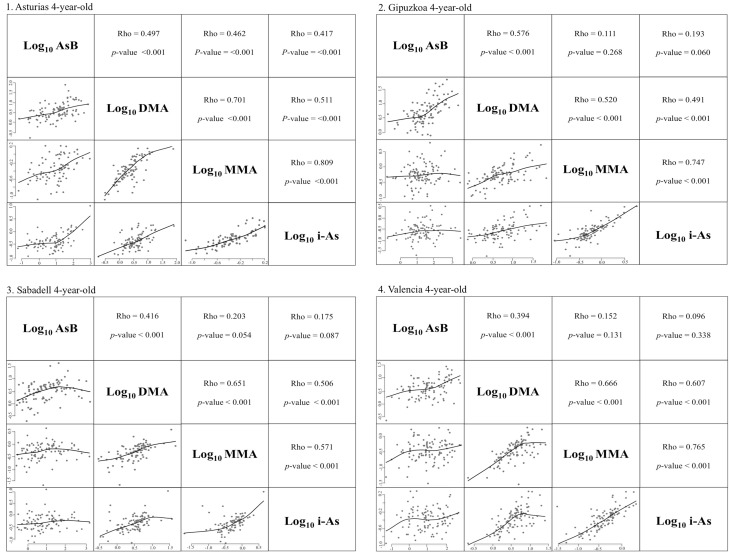
Correlation between arsenic species in urinary 4-year-old samples from each cohort study

Spearman correlation between log_10_ i-As and log_10_ MMA concentration in urine samples showed very strong correlation in Asturias (Rho = 0.809), strong correlation in Gipuzkoa (Rho = 0.747) and Valencia (Rho = 0.765) and moderate correlation in Sabadell (Rho = 0.571). A strong correlation was found between log_10_ i-As and log_10_ DMA in samples from Valencia (Rho = 0.607) compared to the other regions that had a moderate correlation with a Spearman coefficient ranging from 0.491 to 0.511. There was a strong correlation between log_10_ MMA and log_10_ DMA in samples from Asturias (Rho = 0.707), Sabadell (Rho = 0.651) and Valencia (Rho = 0.666) compared to a moderate one in Gipuzkoa (Rho = 0.520). A moderate correlation was found in urine samples from all regions between log_10_ AsB and log_10_ DMA. There were no significant correlations in log_10_ AsB versus log_10_ i-As and log_10_ AsB versus log_10_ MMA in Gipuzkoa, Sabadell and Valencia; on the contrary, a moderate correlation was shown between those urinary As metabolites in samples from Asturias (Fig. 2).

### Pregnant Women–Children of 4- and 7-Year-Old Pairs

**Fig. 3:**
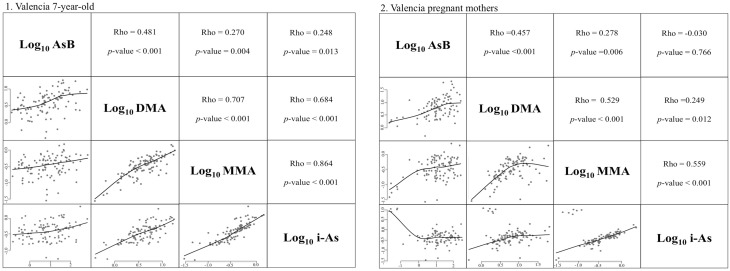
Correlation between arsenic species in urinary pregnant women and 7-year-old children samples from Valencia

DMA concentration was higher in urine samples of pregnant women (median: 5.69 μg/l and IQR: 2.93–10.89 μg/l; *p* < 0.003) than in the urine samples of their children of 4 and 7 years old. There were no statistically significant differences in AsB, MMA and i-As concentration in urine samples between pregnant women and their children of 4 and 7-years old (Table 1). The correlation between log_10_ i-As and log_10_ MMA in 7-year-old children from Valencia was high (Rho = 0.864), a bit lower when they were 4 years old (Rho = 0.765). The correlation between the other As species was similar in urine samples from Valencian children of 4 and 7 years old. A cluster of 6 maternal urine samples with much higher levels of i-As, ranging from 8.76 to 12.86 μg/l, was found, which affected the correlation between log_10_ i-As and the other As species. Despite this cluster of urine samples from pregnant women with higher i-As levels, good correlation was found between log_10_ i-As and log_10_ MMA (Rho = 0.559), which was higher than that found in the 4- and 7-year-old children urine samples when that cluster of samples was not included in the Spearman correlation analysis (Rho = 0.875). There was a moderate correlation between log_10_ MMA and log_10_ DMA in pregnant women urine samples (Rho = 0.529). A moderate correlation was also found between log_10_ i-As and log_10_ DMA in pregnant women urine samples when the cluster of samples with high levels of i-As was not included in the Spearman correlation analysis (Rho = 0.475), which was lower than that found for the 4- and 7-year-old children (Fig. 3).

## Discussion

Almost all pregnant women, 4- and 7-year-old children urinary samples contained detectable (i.e. > LOD) AsB (99, 100 and 100 %, respectively), MMA (95, 93 and 100 %, respectively), DMA (99, 98 and 100 %, respectively) and i-As (100, 96 and 99 %, respectively). Since seafood is the main source of AsB and the presence of this organic As compound in the urine samples suggests that seafood is in the diet of the pregnant women and children, the consumption of which has been estimated to be 26.40 kg/person/year in Spain (Magrama 2015). AsB is excreted unchanged in the urine, while arsenosugars and arsenolipids, also found in seafood, are metabolised predominantly to DMA before being excreted (Choi et al. 2010; Navas-Acien et al. 2011). Each autonomous regional public health administration in Spain monitors the quality of drinking water and there are no reported cases of contamination with i-As in the geographical areas where this study was carried out. Therefore, the i-As levels in drinking water are assumed to be below the EU regulation—10 μg/l (The Council of the European Union 1998). It is expected that i-As exposure comes largely from foodstuffs such as rice and rice-based products due to their high i-As content and regular consumption by the Spanish population (Carbonell-Barrachina et al. 2012; Food and Authority 2014; Meharg et al. 2014; Magrama 2015; Signes-Pastor et al. 2016a). The metabolism of i-As in the human body comprises a series of reduction and methylation reactions, resulting in the formation of MMA (10–20 %) and DMA (60–70 %), which are excreted in urine together with unchanged i-As (10–30 %) (Vahter 2002). DMA is, therefore, a metabolite that reflects exposure of both i-As and organic compounds (Navas-Acien et al. 2011). DMA may also be excreted in the urine unchanged after rice consumption because significant levels of DMA have been reported in rice from certain areas, including rice from Spain (Meharg et al. 2009; Signes-Pastor et al. 2016a). Indeed, 90 % of urinary As excretion after a rice diet containing both i-As and DMA at a 1:1 ratio has been reported to be DMA (Meharg et al. 2014). In general terms, biomethylation is considered the major detoxification process. However, the intermediate metabolites MMA^III^ and DMA^III^ are highly toxic and considered to be toxic species of ingested i-As (Bredfeldt et al. 2006; López-Carrillo et al. 2014).

The sum of arsenic species concentration in urine samples of pregnant women in their 1st trimester of pregnancy found here for the Valencia cohort was lower than the total As level reported previously for pregnant women in their 1st trimester of pregnancy from the Sabadell cohort, for whom seafood was identified as the main source of As (Forns et al. 2014, 2014a, 2014b). However, the urinary arsenic concentration (i-As + MMA + DMA) in pregnant women, found in this study, was almost double than that found in the urine of pregnant women in their 2nd trimester of pregnancy living in the state of New Hampshire in the US (Gilbert-Diamond et al. 2016). DMA concentration in pregnant women was 1.4-fold higher than that found in their children’s urinary samples of both 4- and 7-year-olds, which could be justified with a major exposure to dietary DMA or due to the detoxification process, which has been associated with a higher methylation capacity for women in the early stages of pregnancy (Concha et al. 1998; Vahter 2009). The urinary summation of the As species for 4- and 7-year-old children found in this study was much higher compared to the median of total As previously reported for 6- to 9-year-old children and 5- to 17-year-old children (1.17 and 1.72 μg/l, respectively) living in the Southwest Spain (Huelva, Andalusia) (Aguilera et al. 2010; Rodríguez-Barranco et al. 2016). Children aged 5–8 years from Montevideo, Uruguay, with low-level As exposure from drinking water (median of 0.45 μg/l), had urinary median i-As, MMA and DMA values of 1.01, 0.95 and 7.9 μg/l, respectively (Kordas et al. 2016), which are about double the concentration found in this study. A similar level of urinary i-As to that found here has been reported for native and immigrant children aged 2 months–16 years living in Barcelona, Spain (median 0.4 μg/l) (Piñol et al. 2015). There were no significant differences in any of the urinary As metabolites in relation to sex in keeping with previous studies that did not find sex differences in total urinary As concentration (Aguilera et al. 2010; Rodríguez-Barranco et al. 2016).

All 4-year-old children cohorts had strong methylation capacity to methylate i-As to MMA, especially those from Asturias, Gipuzkoa and Valencia that had 1.35-fold stronger correlation coefficients on average between log_10_ i-As and log_10_ MMA than that for those from Sabadell. The correlation coefficients between log_10_ i-As and log_10_ MMA for 7-year-old children from Valencia were 1.13-fold higher compared to themselves at 4-year-old, which may suggest an increase of capacity to methylate i-As to MMA with age. Indeed, the pregnant women had the highest correlation coefficient between log_10_ i-As and log_10_ MMA when the cluster of samples with high levels of i-As was not included in the calculations. Although lower capacity to further methylate MMA to DMA is suggested by the inferior correlation coefficient between log_10_ MMA and log_10_ DMA compared to the coefficient between log_10_ i-As and log_10_ MMA this is difficult to ascertain as both pregnant women and children may have been consuming seafood, which may affect urinary DMA concentration as implied with a moderate correlation coefficient found between log_10_ AsB and log_10_ DMA. Children from Asturias also had a moderate correlation between log_10_ AsB and log_10_ MMA and between log_10_ AsB and log_10_ i-As. This may indicate consumption of seafood products with significant i-As concentration such as some species of brown algae and bivalves (EFSA 2009; Amlund and Sloth 2011).

## Conclusions

In this study, it is shown that AsB, DMA, MMA and i-As are the main As metabolites in urine of pregnant women, 4- and 7-year-old children living in Spain with low-level As exposure from drinking water. Although further studies are required to identify the main dietary sources of As for the Spanish population, it is expected that seafood and rice items contribute significantly to organic As and i-As exposure, respectively. 4-year-old children from Asturias, Sabadell and Valencia had higher urinary i-As than those from Gipuzkoa. There were also significant differences in urinary AsB and DMA among regions. The similar levels of i-As found in urinary samples of pregnant women and their children of 4 and 7 years old suggest a long-term relatively low i-As dietary exposure. Correlation analyses between urinary As metabolites show a strong ability to methylate i-As to MMA, especially for pregnant women and 7-year-old children.

## References

[CR1] Aguilera I, Daponte A, Gil F (2010). Urinary levels of arsenic and heavy metals in children and adolescents living in the industrialised area of Ria of Huelva (SW Spain). Environ Int.

[CR2] Amlund H, Sloth JJ, Nriagu JO (2011). Arsenic exposure from seafood consumption. Encyclopedia of environmental health.

[CR3] Bredfeldt TG, Jagadish B, Eblin KE (2006). Monomethylarsonous acid induces transformation of human bladder cells. Toxicol Appl Pharmacol.

[CR4] Carbonell-Barrachina AA, Wu X, Ramírez-Gandolfo A (2012). Inorganic arsenic contents in rice-based infant foods from Spain, UK, China and USA. Environ Pollut.

[CR5] Carlin DJ, Naujokas MF, Bradham KD (2015). Arsenic and environmental health: state of the science and future research opportunities. Environ Health Perspect.

[CR6] Cascio C, Raab A, Jenkins RO (2011). The impact of a rice based diet on urinary arsenic. J Environ Monit.

[CR7] Choi BS, Choi SJ, Kim DW (2010). Effects of repeated seafood consumption on urinary excretion of arsenic species by volunteers. Arch Environ Contam Toxicol.

[CR8] Concha G, Vogler G, Lezcano D (1998). Exposure to inorganic arsenic metabolites during early human development. Toxicol Sci.

[CR9] Davis MA, Mackenzie TA, Cottingham KL (2012). Rice consumption and urinary arsenic concentrations in U.S. children. Environ Health Perspect.

[CR10] Davis MA, Li Z, Gilbert-Diamond D (2014). Infant toenails as a biomarker of in utero arsenic exposure. J Expo Sci Environ Epidemiol.

[CR11] EFSA (2009). European food safety authority. Scientific opinion on arsenic in food.

[CR12] Farzan SF, Karagas MR, Chen Y (2013). In utero and early life arsenic exposure in relation to long-term health and disease. Toxicol Appl Pharmacol.

[CR13] Farzan SF, Li Z, Korrick SA (2015). Infant infections and respiratory symptoms in relation to arsenic exposure in a U.S. cohort. Environ Health.

[CR14] Food E, Authority S (2014) Dietary exposure to inorganic arsenic in the European population, 1. doi: 10.2903/j.efsa.2014.3597 10.2903/j.efsa.2021.6380 PMC7845508 33537067

[CR15] Forns J, Fort M, Casas M (2014). Exposure to metals during pregnancy and neuropsychological development at the age of 4 years. Neurotoxicology.

[CR16] Fort M, Cosín-Tomás M, Grimalt JO (2014). Assessment of exposure to trace metals in a cohort of pregnant women from an urban center by urine analysis in the first and third trimesters of pregnancy. Environ Sci Pollut Res.

[CR17] Fort M, Grimalt JO, Casas M, Sunyer J (2014). Food sources of arsenic in pregnant Mediterranean women with high urine concentrations of this metalloid. Environ Sci Pollut Res.

[CR18] Gilbert-Diamond D, Cottingham KL, Gruber JF (2011). Rice consumption contributes to arsenic exposure in US women. Proc Natl Acad Sci USA.

[CR19] Gilbert-Diamond D, Emond JA, Baker ER (2016). Relation between in utero arsenic exposure and birth outcomes in a cohort of mothers and their newborns from new hampshire. Environ Health Perspect.

[CR20] Guxens M, Ballester F, Espada M (2012). Cohort profile: the INMA-INfancia y Medio Ambiente-(environment and childhood) project. Int J Epidemiol.

[CR21] IARC (2004) International agency for research on cancer monographs on the evaluation of carcinogenic risks to humans. Some drinking-water disinfectants and contaminants, including Arsenic PMC7682301 15645577

[CR22] Karagas MR, Punshon T, Sayarath V (2016). Association of rice and rice-product consumption with arsenic exposure early in life. JAMA Pediatr.

[CR23] Kippler M, Skröder H, Mosh S (2016). Elevated childhood exposure to arsenic despite reduced drinking water concentrations—a longitudinal cohort study in rural Bangladesh. Env Inter.

[CR24] Kordas K, Queirolo EI, Mañay N (2016). Low-level arsenic exposure: nutritional and dietary predictors in first-grade Uruguayan children. Environ Res.

[CR25] López-Carrillo L, Hernández-Ramírez RU, Gandolfi AJ (2014). Arsenic methylation capacity is associated with breast cancer in northern Mexico. Toxicol Appl Pharmacol.

[CR26] Magrama (2015) Informe del Consumo de Alimentación en España. Ministerio de Agricultura Alimentación y Medio Ambiente

[CR27] Meharg AA, Zhao FJ (2012). Arsenic & rice.

[CR28] Meharg AA, Williams PN, Adomako E (2009). Geographical variation in total and inorganic arsenic content of polished (white) rice. Environ Sci Technol.

[CR29] Meharg AA, Williams PN, Deacon CM (2014). Urinary excretion of arsenic following rice consumption. Environ Pollut.

[CR30] Navas-Acien A, Francesconi KA, Silbergeld EK, Guallar E (2011). Seafood intake and urine concentrations of total arsenic, dimethylarsinate and arsenobetaine in the US population. Environ Res.

[CR31] Piñol S, Sala A, Guzman C (2015). Arsenic levels in immigrant children from countries at risk of consuming arsenic polluted water compared to children from Barcelona. Environ Monit Assess.

[CR32] R Core Team (2014). R: A language and enrionment for statistical computing.

[CR33] Rodríguez-Barranco M, Gil F, Hernández AF (2016). Postnatal arsenic exposure and attention impairment in school children. Cortex.

[CR34] Sanchez TR, Perzanowski M, Graziano JH (2016). Inorganic arsenic and respiratory health, from early life exposure to sex-specific effects: a systematic review. Environ Res.

[CR35] Signes-Pastor AJ, Carey M, Carbonell-Barrachina AA (2016). Geographical variation in inorganic arsenic in paddy field samples and commercial rice from the Iberian Peninsula. Food Chem.

[CR36] Signes-Pastor AJ, Carey M, Meharg AA (2016). Inorganic arsenic in rice-based products for infants and young children. Food Chem.

[CR37] Sohn E (2014) The toxic side of rice. Nat Outlook, pp 5–6 10.1038/514s62a 25368891

[CR38] The Council of the European Union (1998). Council directive 98/83/EC of 3 November 1998 on the quality of water intended for human consumption. Off J Eur Commun L.

[CR39] Vahter M (2002). Mechanisms of arsenic biotransformation. Toxicology.

[CR40] Vahter M (2009). Effects of arsenic on maternal and fetal health. Annu Rev Nutr.

